# Cuproptosis-driven astrocyte reactivity exacerbates experimental cerebral malaria pathogenesis

**DOI:** 10.1186/s13071-025-07107-0

**Published:** 2025-11-10

**Authors:** Xinpeng Hou, Xiumei Mo, Xiaoran Zhang, Qi Wang, Xiaoyan Chen, Chufang Lai, Jiamei Gao, Lirong Wu, Wenbin Liu, Jiajing He, Xingda Zeng, Hui Yin, Zujun Deng, Tao Liu, Minqiu Ye, Zhenlong Liu, Xiaobao Jin, Jianping Song, Jie Wang, Bo Huang

**Affiliations:** 1 Guangdong Provincial Key Laboratory of Pharmaceutical Bioactive Substances, Guangdong Pharmaceutical University, Guangzhou, 510006 People’s Republic of China; 2 Department of Clinical Laboratory, Shenzhen Traditional Chinese Medicine Hospital, The Fourth Clinical Medical College of Guangzhou University of Chinese Medicine, Shenzhen, 518033 People’s Republic of China; 3 Guangzhou Chest Hospital, Guangzhou, 510095 People’s Republic of China; 4 Guangzhou Olympic High School, Guangzhou, 510660 People’s Republic of China; 5 Laboratory Animal Center, Guangdong Pharmaceutical University, Guangzhou, 510006 People’s Republic of China; 6 Department of Critical Care Medicine, Foshan Sanshui District People’s Hospital, Foshan, 528199 People’s Republic of China; 7 Division of Experimental Medicine, Department of Medicine, McGill University, Montreal, QC Canada; 8 Artemisinin Research Center, Guangzhou University of Chinese Medicine, Guangzhou, 510405 People’s Republic of China

**Keywords:** Cerebral malaria, Blood–brain barrier, Astrocyte, Cuproptosis

## Abstract

**Background:**

Cerebral malaria (CM), a lethal neurological complication of *Plasmodium falciparum*, is characterized by blood–brain barrier (BBB) disruption. Although astrocytes constitute essential components of the BBB neurovascular unit, their immunoregulatory functions during CM pathogenesis remain elusive. Clinical evidence of altered copper homeostasis in patients with CM, coupled with known associations between copper dysregulation and astrocyte reactivity, prompted investigation of cuproptosis—a copper-dependent programmed cell death pathway—in the disease progression of CM.

**Methods:**

Using a *P. berghei* ANKA (*Pb*A)-induced experimental CM (ECM) model in C57BL/6 mice, we evaluated pharmacological modulation with copper ionophore disulfiram (DSF) versus copper chelator tetrathiomolybdate (TTM). Parallel in vitro experiments assessed astrocytes stimulated by *Pb*A-infected red blood cells (iRBCs)/blood-stage soluble antigen (*Pb*Ag) under DSF-CuCl_2_ or TTM-CuCl_2_ treatment.

**Results:**

ECM mice demonstrated significant cerebral copper accumulation with concomitant upregulation of cuproptosis markers (SLC31A1, FDX1, DLAT, and DLST) and downregulation of ATP7A copper transporter. DSF administration exacerbated ECM progression through amplified parasitemia, aggravated BBB permeability, cerebral edema, and neuroinflammatory responses, whereas TTM treatment counteracted these pathological manifestations. Immunohistochemical analysis revealed DSF-induced astrocyte reactivity (GFAP^+^/Serping1^+^) with colocalization of cuproptosis markers (GFAP^+^-SLC31A1^+^/FDX1^+^/DLAT^+^/DLST^+^), contrasting with TTM-mediated suppression. In vitro, DSF-CuCl_2_ treatment augmented iRBC-stimulated astrocyte expression of reactivity markers (GFAP and Serping1), cuproptosis regulators (SLC31A1, FDX1, DLAT, and DLST), and proinflammatory mediators (CXCL10, tumor necrosis factor (TNF)-ɑ, interleukin (IL)-1β, and IL-6), but conversely reduced *Pb*Ag-stimulated cell viability. These effects were reversed by TTM-CuCl_2_ treatment.

**Conclusions:**

These findings establish that cuproptosis exacerbates ECM pathogenesis by promoting astrocyte reactivity, highlighting copper homeostasis modulation as a potential therapeutic strategy for CM.

**Graphical Abstract:**

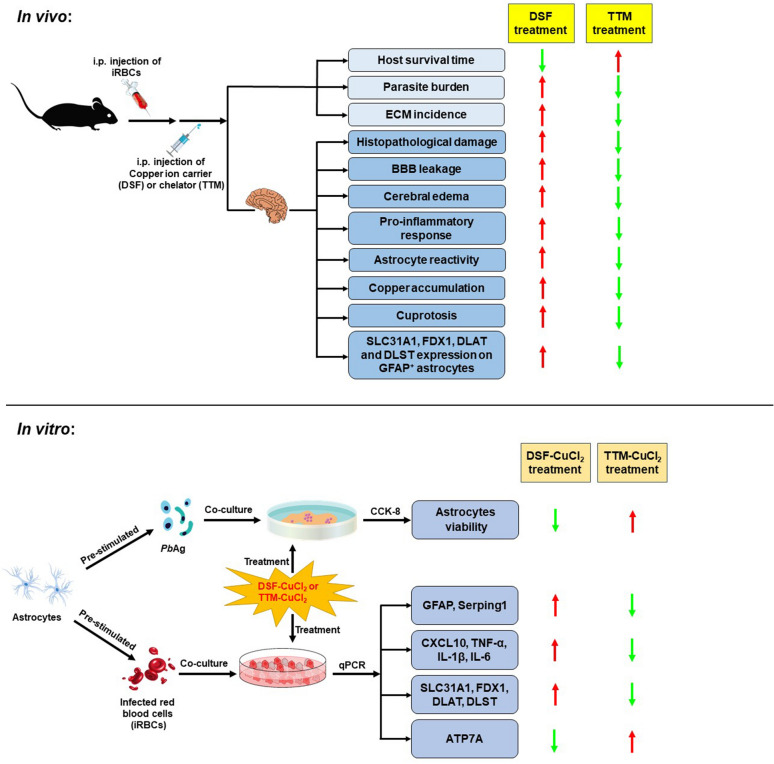

**Supplementary Information:**

The online version contains supplementary material available at 10.1186/s13071-025-07107-0.

## Background

Malaria, a life-threatening disease caused by *Plasmodium* parasites, continues to impose a substantial global burden with 263 million clinical cases and 597,000 fatalities reported in 2023, predominantly affecting children under 5 years in sub-Saharan Africa [1]. *Plasmodium falciparum* infection accounts for over 90% of severe malaria manifestations, including cerebral malaria (CM)—a rapidly progressing neurological syndrome characterized by impaired consciousness, seizures, and persistent neurocognitive deficits [2–4]. Despite advances in antimalarial chemotherapy, CM mortality remains alarmingly high (15–20%), with 25% of survivors experiencing long-term neurological complications, highlighting urgent needs for novel therapeutic strategies targeting pathogenic mechanisms [5]. The pathophysiology of CM involves a cascade of events initiated by sequestration of infected erythrocytes (iRBCs) in cerebral microvasculature, leading to endothelial activation, blood–brain barrier (BBB) breakdown, and maladaptive neuroimmune responses [6–8]. Astrocytes, comprising 20–40% of human brain cellular composition, serve as crucial regulators of BBB integrity, cerebral metabolic homeostasis, and inflammatory modulation through dynamic neuron-glial interactions [9, 10]. While early pioneering studies proposed distinct neurotoxic or neuroprotective phenotypes for reactive astrocytes [11, 12], recent advances underscore their dynamic phenotypic heterogeneity, shaped by disease-specific microenvironments and temporal dynamics, ultimately dictating functional outcomes in neuropathological contexts [13–16]. While reactive astrocytes have been clinically correlated with BBB dysfunction in both human CM autopsy specimens and experimental CM (ECM) murine models [17–19], preclinical studies demonstrate that pharmacological suppression of astrocytic reactivity via intravenous artesunate administration attenuates hippocampal neurodegeneration and improves ECM outcomes [20]. Although the current understanding emphasizes parasite-derived metabolites (e.g., heme-induced oxidative stress) as primary drivers of endothelial dysfunction in CM pathogenesis, recent findings suggest that astrocyte-mediated neuroinflammation may critically influence long-term neurological sequelae. However, the role of reactive astrocytes in modulating CM-related neuroinflammatory cascades remains incompletely defined.

Copper homeostasis, a tightly regulated process encompassing absorption, distribution, and excretion, is essential for maintaining systemic physiological balance [21]. Within the central nervous system (CNS), copper plays pivotal roles in neurodevelopment, synaptic plasticity, and antioxidant defense; cerebral copper concentrations ranking third among human organs, accounting for ~9% of total body reserves [22]. The blood–brain barrier (BBB) precisely governs central nervous system (CNS) copper dynamics through coordinated action of copper transporters (CTR1 and ATP7A), while simultaneously preventing systemic copper overload via ATP7B-mediated efflux [23]. Dyshomeostasis of this regulatory network underlies neuropathologies spanning Menkes disease to Alzheimer’s and Huntington’s diseases [24]. Crucially, copper exerts dual biological effects: as a cofactor for cytochrome c oxidase and superoxide dismutase at physiological levels, but triggering cuproptosis—uproptosis superoxide dismutase at pathological levels [25]. Astrocytes, the primary CNS metalloregulators, maintain copper equilibrium through CTR1-mediated uptake and metallothionein-dependent storage [26]. Recent mechanistic studies define cuproptosis as FDX1-dependent lipoylated protein aggregation (DLAT/DLST) with subsequent proteotoxic stress, distinct from other cell death pathways [25]. Clinically, elevated serum copper correlates with severe malaria mortality [27, 28], while copper chelators demonstrate antimalarial efficacy through *Plasmodium* growth inhibition [29, 30]. Experimental models reveal cuproptosis contributes to cognitive impairment via CREB–BDNF pathway suppression [31], with transcriptomic signatures linking cuproptosis regulators (ATP7A, FDX1, and NLRP3) to neuroinflammatory cascades in stroke and Parkinson’s disease [32, 33]. Notably, astrocytes exhibit particular vulnerability to copper toxicity owing to their high metabolic demands and antioxidant synthesis functions [34]. Despite these advances, the potential interplay between copper overload-induced astrocytic cuproptosis and cerebral malaria-associated neurovascular pathology—particularly in BBB disruption and neuroinflammation driven by reactive astrocytes—remains unexplored, representing a critical knowledge gap in CM pathogenesis.

To elucidate the role of cuproptosis in CM pathogenesis, we established an ECM model by infecting C57BL/6 mice with *P. berghei* ANKA (*Pb*A) strain. Pharmacological modulation was achieved through intraperitoneal administration of disulfiram (DSF; a copper ionophore) or tetrathiomolybdate (TTM; a copper chelator) to ECM mice, targeting cerebral copper homeostasis. Complementary in vitro studies employed a coculture system of iRBCs/blood-stage soluble antigen (*Pb*Ag)-stimulated astrocytes with DSF-CuCl_2_ or TTM-CuCl_2_ to dissect molecular mechanisms. Key findings revealed that cuproptosis critically contributes to ECM progression. Notably, DSF exacerbated cerebral cuproptosis, potentiated inflammatory astrocyte reactivity, and aggravated severity of ECM. In stark contrast, TTM suppressed cuproptosis, attenuated astrocyte reactivity, and improved survival outcomes. These bidirectional effects were corroborated in vitro*,* where DSF-CuCl_2_ amplified proinflammatory cytokine expression in iRBCs-stimulated astrocytes, but reduced *Pb*Ag-stimulated cell viability. While TTM-CuCl_2_ treatment reversed these effects. Collectively, our study provides the novel experimental evidence linking astrocytic cuproptosis to ECM neuropathology and identifies copper homeostasis modulation as a translational strategy for mitigating CM sequelae.

## Methods

### Mice, parasites, and ethics

Female C57BL/6 mice (6 weeks old, specific pathogen free (SPF)-grade) were sourced from the Guangdong Provincial Medical Laboratory Animal Center and housed under SPF conditions at Guangdong Pharmaceutical University’s Laboratory Animal Center, with controlled environmental parameters (12-h light/dark cycle; 22–25 °C; 45 ± 5% humidity). The *Plasmodium berghei* ANKA (*Pb*A) strain, cryopreserved in liquid nitrogen postrecovery from infected mouse blood, was thawed at 37 °C. To maintain parasite viability and genetic stability, cryopreserved *Pb*A-infected red blood cells [iRBCs; 1 × 10^6^ cells in 200 μL phosphate buffered saline (PBS)] were intraperitoneally (i.p.) injected into 6-week-old female C57BL/6 donor mice. Peripheral parasitemia in donor mice was monitored via Giemsa-stained thin blood smears, and when levels reached approximately 10%, iRBCs were transferred i.p. (1 × 10^6^ cells in 200 μL PBS) to naive recipient mice for sequential amplification and downstream experiments. All procedures adhered to the Chinese Regulations for the Administration of Laboratory Animals and were approved by the Animal Ethics Committee of Guangdong Pharmaceutical University (Ethics Approval No. GDPULAC2024026).

### DSF or TTM administration to *Pb*A-infected mice

A total of 156 C57BL/6 mice were randomly allocated into six experimental groups: naive (*n* = 24), DSF (*n* = 24), TTM (*n* = 24), *Pb* (*n* = 28), *Pb* + DSF (*n* = 28), and *Pb* + TTM (*n* = 28). Noninfected groups (naive, DSF, and TTM) received daily intraperitoneal (i.p.) injections of 100 μL PBS, DSF (50 mg/kg; Sigma-Aldrich 86720), or TTM (30 mg/kg; Sigma-Aldrich 323446), respectively, until experimental termination. Infection groups (*Pb*, *Pb* + DSF, and *Pb* + TTM) were inoculated i.p. with 1.0 × 10^6^ *Pb*A-iRBCs, followed by daily i.p. administration of PBS, DSF (50 mg/kg), and TTM (30 mg/kg) from day 1 postinfection (p.i.), respectively. Mice displaying neurological manifestations (ataxia, coma, convulsions, or paralysis) accompanied by mortality within 24 h during days 6–9 p.i. were classified as ECM cases. Survival monitoring and neurological symptom monitoring were conducted daily. Concurrently, parasitemia progression was quantified through Giemsa-stained thin blood smears from tail veins (× 1000 magnification), calculated as: (iRBCs count/total RBCs count) × 100%, until study completion. Notably, uninfected mice from the naive, DSF, and TTM groups, along with ECM mice reaching terminal morbidity between days 6–9 post-infection (p.i.) from *Pb*, *Pb* + DSF, and *Pb* + TTM-infected groups, were randomly selected for experimental analyses in Sect. Histopathological analysis–Quantitative real-time PCR analysis of astrocytic reactivity and cuproptosis markers.

### Histopathological analysis

To evaluate DSF or TTM effects on neuropathology in *Pb*A-uninfected/infected mice, hematoxylin and eosin (H and E) staining was performed. Six mice per group were randomly euthanized, and brain tissues were collected, paraffin-embedded, and sectioned into 5 μm-thick slices. Three nonconsecutive sections per animal underwent deparaffinization, rehydration, and H and E staining. Blinded histopathological assessment was conducted by two independent pathologists using a Leica DM2500B microscope (400 × magnification), with > 20 nonoverlapping fields analyzed per section. Pathological features, including hemorrhagic foci, were quantified according to established scoring criteria [35].

### Assessment of BBB integrity

BBB permeability was evaluated using Evans Blue extravasation. Six mice per group were intravenously administered Evans Blue (2% in PBS, 4 mL/kg) on day 6 postinfection (p.i.) following established protocols [36]. At 30 min postinjection, animals were euthanized via transcardial perfusion with 20 mL PBS followed by 4% paraformaldehyde to clear intravascular dye. Brains were dissected, homogenized in 50 mL formamide (60 °C 24 h), and centrifuged. Evans Blue concentration in supernatants was quantified spectrophotometrically at 620 nm (iMark microplate reader, Bio-Rad), with tissue dry weights determined after overnight at 80 °C. Dye extravasation was calculated against a standard curve and normalized as ng Evans Blue/mg dry tissue mass.

### Measurement of cerebral edema

Cerebral edema was quantified through gravimetric analysis. Mice (*n* = 6/group) were euthanized on day 6 post-infection, and brains were immediately excised. Posteuthanasia, tissues were blotted dry and weighed to determine wet weight (WW), then dehydrated at 80 °C for 18 h to obtain dry weight (DW). Edema severity was calculated as: [(WW–DW)/WW] × 100%.

### Cerebral copper quantification

Copper deposition in cerebral tissues was assessed through rubeanic acid copper staining and inductively coupled plasma mass spectrometry (ICP-MS) analyses. Paraffin-embedded brain sections (*n* = 6 mice/group) underwent rubeanic acid copper (RAC) staining using three nonconsecutive sections per mouse. Postdeparaffinization and hydration, tissues were stained with 0.1% rubeanic acid (60 min, 37 °C), counterstained with nuclear fast red, and mounted. Dark green-black copper granules were quantified via integrated optical density (IOD/μm^2^) across > 20 nonoverlapping fields per section (Leica DM IRE2, × 200 magnification), with blinded image acquisition using a DM 2500B microscope (× 400). For inductively coupled plasma mass spectrometry (ICP-MS), tissues were microwave-digested sequentially in MOS-grade HNO_3_ (65 °C, 2 h) and HClO_4_ (120 °C, 6 h), re-dissolved in 4% HNO_3_ (60 °C, 1 h), and centrifuged (12,000 × *g*, 10 min). Copper concentrations were taken as μg copper/g wet weight of brain.

### Immunohistochemical staining for GFAP, Serping1, SLC31A1, ATP7A, FDX1, DLAT, DLST, and S100B in brain tissues of ECM mice

Three nonconsecutive paraffin-embedded brain sections per mouse (*n* = 6/group) were processed through standardized immunohistochemical workflows. Following deparaffinization in xylene and rehydration through graded ethanol series (100–70%), antigen retrieval was performed in preheated 10 mM sodium citrate buffer (pH 6.0, 95 °C, 10 min). Endogenous peroxidase activity was quenched with 3% H_2_O_2_ (37 °C, 25 min, dark incubation), followed by PBS washes (3 × 5 min) and blocking with 10% normal goat serum (RT, 30 min). Sections were incubated with primary antibodies at 4 °C for overnight: GFAP (1:3,000; GB11096; Servicebio), Serping1 (1:1,000; GB112165; Servicebio), SLC31A1 (1:250; NB100-402; Novus), ATP7A (1:100; MA5-27720; Thermo Fisher), FDX1 (1:200; 12592–1-AP; Proteintech), DLAT (1:1,000; GB113649; Servicebio), DLST (1:1,500; GB114020; Servicebio), and S100B (1:500; GB15359; Servicebio). After PBS rinsing, horseradish peroxidase (HRP)-conjugated secondary antibodies (1:200; Affinity S0001/S0002) were applied (RT, 60 min), followed by 3,3′-diaminobenzidine (DAB) chromogenic development (2 min) and hematoxylin counterstaining. Blinded quantification was performed by two investigators using a Leica DM 2500B microscope (× 400) with > 20 nonoverlapping fields/section. Astrocytic markers GFAP and Serping1 were quantified as integrated optical density/area (IOD/μm^2^), while copper transporters (SLC31A1, ATP7A), cuproptosis regulators (FDX1, DLAT, DLST), and S100B (Astrocytic markers damage marker) were scored as positive cells/field (0.015 mm^2^).

### Double immunofluorescence staining for GFAP^+^-SLC31A1^+^, GFAP^+^-FDX1^+^, GFAP^+^-DLAT^+^, and GFAP^+^-DLST^+^ astrocytic cuproptosis in brain tissue of ECM mice

Sequential double immunofluorescence staining was performed to assess expression of cuproptosis regulators (SLC31A1, FDX1, DLAT, and DLST) within GFAP^+^ astrocytes. Three nonadjacent paraffin sections/mouse (*n* = 6/group) underwent xylene deparaffinization followed by graded ethanol rehydration (100–70%). Antigen retrieval was achieved using preheated 10 mM sodium citrate buffer (pH 6.0, 95 °C, 10 min). Endogenous peroxidase activity was suppressed with 3% H_2_O_2_ (37 °C, 25 min, dark incubation), followed by three PBS washes (5 min each) and blocking with 10% normal goat serum (RT, 30 min). Primary antibodies were applied in pairwise combinations for overnight at 4 °C: mouse anti-GFAP (1:2,500; GB12100, Servicebio) with rabbit anti-SLC31A1 (1:500; NB100-402, Novus), anti-FDX1 (1:200; 12592-1-AP, Proteintech), anti-DLAT (1:500; GB113649, Servicebio), or anti-DLST (1:1,000; GB114020, Servicebio). After PBS rinsing, sections were incubated with species-matched secondary antibodies—Alexa Fluor^®^ 488-conjugated anti-mouse (1:1,000; 4480S, CST) and CoraLite^®^ 594-conjugated anti-rabbit (1:200; SA00013-4, Proteintech)—for 60 min at 37 °C (dark conditions). Nuclei were counterstained with DAPI (10 min, RT), with fluorescence imaging conducted using an EVOS M5000 system (Thermo Fisher) at 488 nm (GFAP), 594 nm (target proteins), and 358 nm (DAPI). Colocalization events (yellow signal) were quantified across > 20 nonoverlapping fields/Sect. (0.015 mm^2^/field, × 400) by two blinded investigators, expressed as double-positive cells/field.

### In vitro astrocyte cell viability assay

To evaluate the effect of cuproptosis on astrocytes viability, iRBCs-stimulated astrocytes were cocultured with DSF or TTM and Cell Counting Kit-8 assay (CCK-8) assay was performed. Preparation of *Pb*ANKA blood-phase soluble antigen was conducted as previously described [35]. Primary cortical astrocytes were isolated from postnatal day 1–3 C57BL/6 mice under aseptic conditions. Neonates were euthanized by decapitation, followed by cerebellar and meningeal removal before cortical dissection. Tissue dissociation was achieved through 0.25% trypsin (EDTA-free; Gibco) digestion (37 °C, 15 min), filtration through 70 μm nylon meshes (BD Falcon), and centrifugation (200 × *g*, 8 min). Pelleted cells were resuspended in Dulbecco’s modified eagle medium (DMEM)/F-12 medium supplemented with 20% fetal bovine serum (FBS; Gibco) and plated on poly-L-lysine-coated flasks at 1 × 10^6^ cells/mL. Cultures were maintained in 10% FBS/DMEM/F-12 under standard conditions (37 °C, 5% CO_2_, 95% humidity) with medium renewal every 3 days. The harvested astrocytes were resuspended, adjusted to a 1 × 10^4^ cells/mL, seeded into 96-well plates. The cells were then subjected to one of the following equal volume of complete medium treatments for 12, 24, or 48 h: (1) *Pb*Ag (20 μg/mL), (2) *Pb*Ag (20 μg/mL) + DSF (20 nM) + CuCl_2_ (10 μM), or (3) *Pb*Ag (20 μg/mL) + TTM (20 nM) + CuCl_2_ (10 μM). Astrocytes treated with PBS alone served as the control (or naive) group. After treatment, 10 μL CCK-8 (Beyotime Biotechnology, China) was added to each well, followed by incubation for 1 h at 37 °C. The absorbance of each well was measured at 450 nm (iMark microplate reader, Bio-Rad).

### In vitro coculture experiment of iRBCs-stimulated astrocytes with DSF-CuCl_2_ or TTM-CuCl_2_

To further evaluate the effect of DSF or TTM on the expression of cuproptosis-related-genes, astrocyte reactivity, and the release of cyokines, a coculture experiment of iRBCs-stimulated astrocytes with DSF-CuCl_2_ or TTM-CuCl_2_ was conducted. For experiments, confluent astrocytes (passage 2–3) were seeded in 6-well plates (2 × 10^5^ cells/well), allowed to adhere for 6 h, then stimulated with 2 × 10^6^ RBCs/well or 2 × 10^6^ iRBCs/well. After 24 h priming, iRBCs-stimulated astrocytes were then subjected to one of the following equal volume treatments for 24 or 48 h: (1) DSF (20 nM) + CuCl_2_ (10 μM), or (2) TTM (20 nM) + CuCl_2_ (10 μM) for 24/48 h. Astrocytes treated with PBS alone served as the control (or naive) group. Post-treatment, cells were washed thrice with ice-cold PBS (pH 7.4), lysed with TRIzol™ (TaKaRa, #9109), and stored at −80 °C until RNA extraction.

### Quantitative real-time PCR analysis of astrocytic reactivity and cuproptosis markers

Total RNA was isolated from cortical brain tissues and primary astrocyte cultures at different groups using TRIzol™ (Takara Bio, #9109). RNA integrity was verified by A260/A280 ratios (1.8–2.0; NanoDrop 2000, Thermo Fisher) and reverse-transcribed using the PrimeScript™ 1st Strand cDNA Synthesis Kit (Takara Bio, #6210B) under standardized conditions: 42 °C for 30 min, 85 °C for 5 min. SYBR Green-based qPCR amplification was performed using SYBR Green qPCR Master Mix (6110B#, TaKaRa) in 20 μL reactions (CFX96 Touch System, Bio-Rad) with the following cycling parameters: 95 °C for 30 s (initial denaturation); 45 cycles of 95 °C for 5 s, 60 °C for 30 s (annealing/extension). Primer pairs (Supplementary Table 1) were designed using Primer-BLAST (NCBI) to span exon–exon junctions (Sangon Biotech, Shanghai), with melting curve analysis confirming single amplicons. Gene expression quantification (GFAP, Serping1, CXCL10, TNF-α, IL-1β, IL-6, SLC31A1, ATP7A, FDX1, DLAT, DLST, and *Pb*A 18S rRNA) was normalized to β-actin using the 2^−ΔΔCt^ method.

### Statistical analysis

All quantitative data were analyzed using GraphPad Prism v8.0.4 (GraphPad Software). Continuous variables were presented as mean ± SD. Two-group comparisons with equal variance employed unpaired two-tailed Student’s *t *tests. Multi-group comparisons utilized one-way analysis of variance (ANOVA) with Tukey’s post-hoc correction. Statistical significance was defined as two-tailed *P* < 0.05.

## Results

### Administration of DSF or TTM altered cerebral parasite burden and ECM incidence in *Pb*A-infected mice

**Fig. 1:**
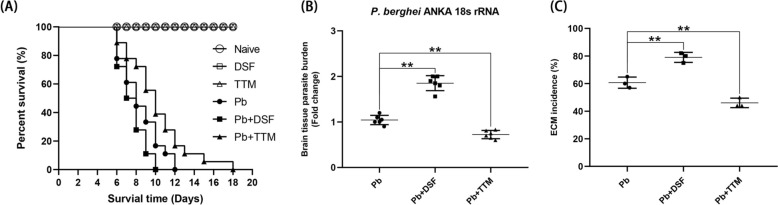
Effects of disulfiram (DSF) or tetrathiomolybdate (TTM) treatment on survival time, cerebral parasite burden, and experimental cerebral malaria (ECM) incidence in *Pb*A-infected mice. **A** Survival time were monitored daily in six groups: **a** naive mice (naive group); **b** DSF-treated uninfected controls (DSF group); **c** TTM-treated uninfected controls (TTM group); **d** untreated *Pb*A-infected mice (*Pb* group); **e** DSF-treated infected mice (*Pb* + DSF group); **f** TTM-treated infected mice (*Pb* + TTM group). **B** Cerebral parasite burden was estimated in ECM mice from the *Pb*, *Pb* + DSF, and *Pb* + TTM groups by quantifying *Pb*A 18S rRNA mRNA levels using qPCR and the 2^−ΔΔCT^ method. **C** ECM incidence was calculated for the *Pb*, *Pb* + DSF, and *Pb* + TTM groups. Data represent mean ± standard deviation from three independent experiments. Statistical significance (^*^ *P* < 0.05, ^**^ *P* < 0.01) was determined relative to *Pb*A infection-induced ECM controls (*Pb* group)

In a controlled survival study (*n* = 12/group), uninfected and *Pb*A-infected C57BL/6 mice received daily i.p. injections of PBS (200 μL), DSF (50 mg/kg), or TTM (30 mg/kg) from day 1 postinfection (p.i.). Survival curves (Fig. 1A) confirmed 100% survival of nonparasitized groups (naive, DSF, TTM) through 18 days, while all *Pb*A-infected cohorts exhibited mortality between days 6–18 p.i.. *Pb*A-infected control mice developed hallmark ECM symptoms including ataxia, hemiplegia, and terminal convulsions, with ~60% mortality by neurological criteria within 24 h of symptom onset. qPCR analysis of brain homogenates revealed divergent treatment effects: DSF administration increased *Pb*A 18S rRNA copies by 1.8-fold versus *Pb*A controls (*P* < 0.01), whereas TTM reduced parasite load by ~60% (*P* < 0.01) (Fig. 1B). It was also demonstrated DSF increased ECM incidence from ~60% (*Pb*A) to ~80% (*P* < 0.01), while TTM reduced incidence to 46% (*P* < 0.01) (Fig. 1C). Mortality kinetics analysis showed DSF accelerated median survival to 8 d versus 11 days in *Pb*A controls (*P* < 0.05), contrasting with TTM-mediated extension to 14 d (*P* < 0.05).

### Effects of DSF or TTM treatment on ECM-associated brain injury

**Fig. 2:**
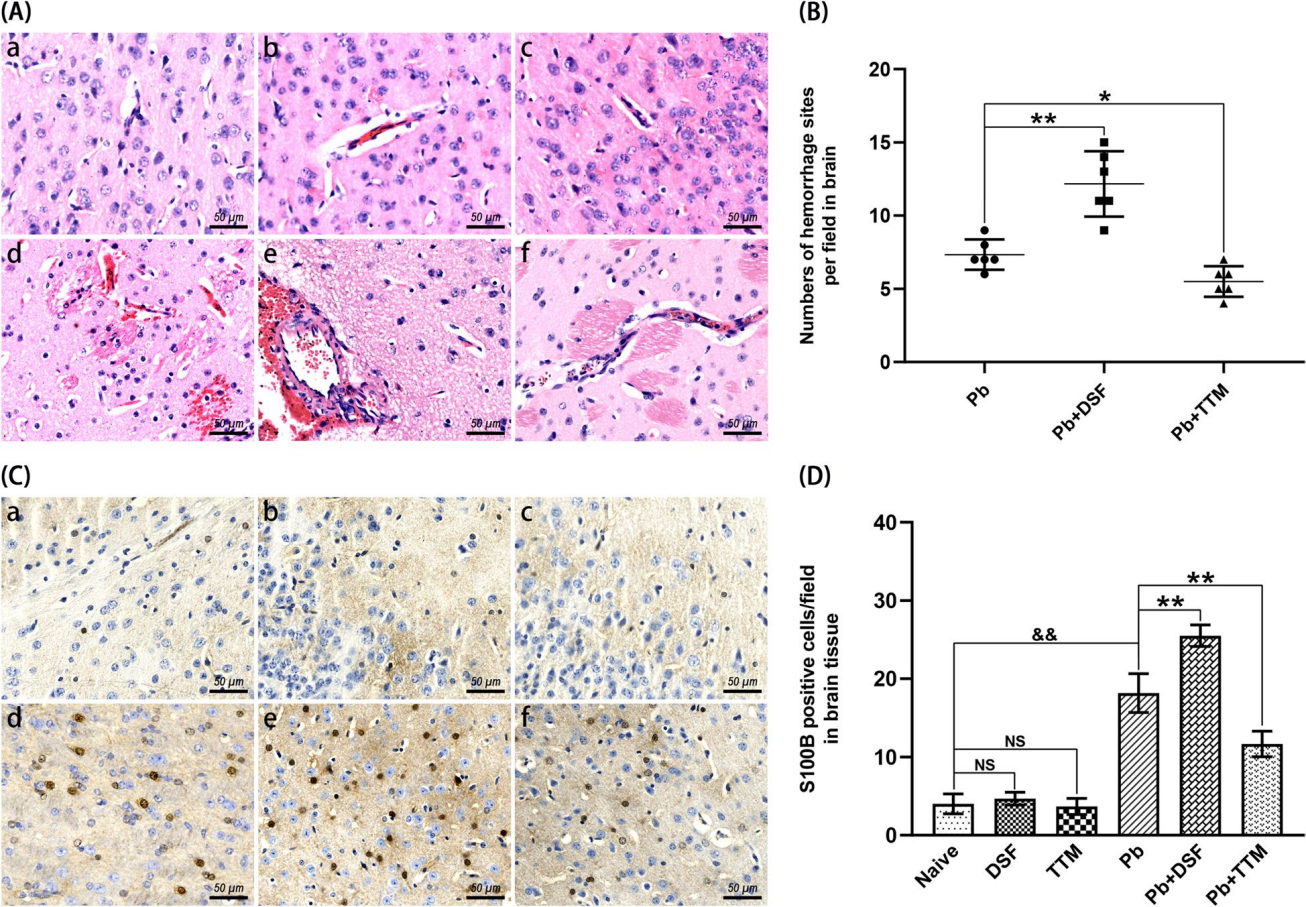
Effects of DSF or TTM treatment on brain injury in ECM mice. **A** Representative hematoxylin and eosin (H and E)-stained brain tissue Sects. (400 × magnification) showing pathological features in six groups: **a** naive mice; **b** DSF-treated uninfected controls; **c** TTM-treated uninfected controls; **d** untreated *Pb*A-infected mice; **e** DSF-treated infected mice; **f** TTM-treated infected mice. **B** Quantitative analysis of hemorrhagic foci per microscopic field (100 × magnification), with > 20 nonoverlapping fields analyzed per section. **C** Immunohistochemical detection of S100B-positive astrocytes (dark brown staining) in cerebral cortex Sects. (400 × magnification) corresponding to groups (**a**-**f**). **D** Semi-quantification of S100B^+^ cells across > 20 cortical fields per group. Data represent mean ± SD (*n* = 6 mice/group, triplicate experiments). Statistical comparisons employed independent *t* tests (two-group) or one-way analysis of variance (ANOVA; multi-group) with post-hoc analysis. Significance thresholds: ^*^P < 0.05, ^**^ P < 0.01 versus infected controls; ^&&^ *P* < 0.01 versus naive mice (group a); NS (not significant, *P* > 0.05) indicates no difference compared with naive mice

To evaluate pathological alterations in brain tissue of *Pb*A infection-induced ECM mice treated with DSF or TTM, H and E staining was performed. As shown in Fig. 2A, naive, DSF-only, and TTM-only control groups exhibited no notable inflammatory cell infiltrates or sequestered iRBCs in cerebral microvasculature. In contrast, ECM mice in the *Pb* group displayed severe neuropathology, including perivascular mononuclear cell infiltration (monocytes/lymphocytes), iRBCs-mediated microvascular obstruction, and multifocal hemorrhages. Strikingly, DSF coadministration with *Pb*A infection (*Pb* + DSF group) exacerbated cerebral damage, demonstrating amplified inflammatory infiltrates, iRBCs sequestration, and hemorrhagic lesions compared with *Pb* group. Conversely, TTM treatment (*Pb* + TTM group) markedly attenuated these pathological features. Quantitative analysis revealed significantly higher hemorrhagic foci density in ECM mice at *Pb* + DSF group versus *Pb* controls (12.2 versus 7.3 foci/field; *P* < 0.01), whereas TTM administration reduced hemorrhage frequency (5.5 versus 7.3 foci/field; *P* < 0.05). These findings demonstrate that DSF potentiates whereas TTM ameliorates *Pb*A-induced cerebral pathology in ECM.

S100B, a biomarker for CNS injury and astrocytic damage, exacerbates neuroinflammation during CM progression by activating microglial and neuronal pathways. To investigate the modulation of S100B-associated brain injury by DSF or TTM, immunohistochemical analysis of cortical brain tissue was conducted in *Pb*A infection-induced ECM mice. As shown in Fig. 2C, sparse S100B-positive cells were detected in cortical regions of naive, DSF-only, and TTM-only control groups. In contrast, ECM mice at *Pb* group exhibited a significant elevation in cortical S100B^+^ cell density compared with uninfected controls (*P* < 0.01). Strikingly, DSF treatment (*Pb* + DSF group) further amplified S100B expression (25.5 versus 18.2 cells/field in *Pb* controls;* P* < 0.01), whereas TTM administration (*Pb* + TTM group) reduced S100B^+^ cell counts to levels below those of the *Pb* group (11.7 versus 18.2 cells/field; *P* < 0.01). These results demonstrate that DSF potentiates *Pb*A infecion-induced neuropathology through S100B upregulation, while TTM confers neuroprotective effects via S100B downregulation.

### Effects of DSF or TTM treatment on BBB integrity and cerebral edema in ECM mice

**Fig. 3:**
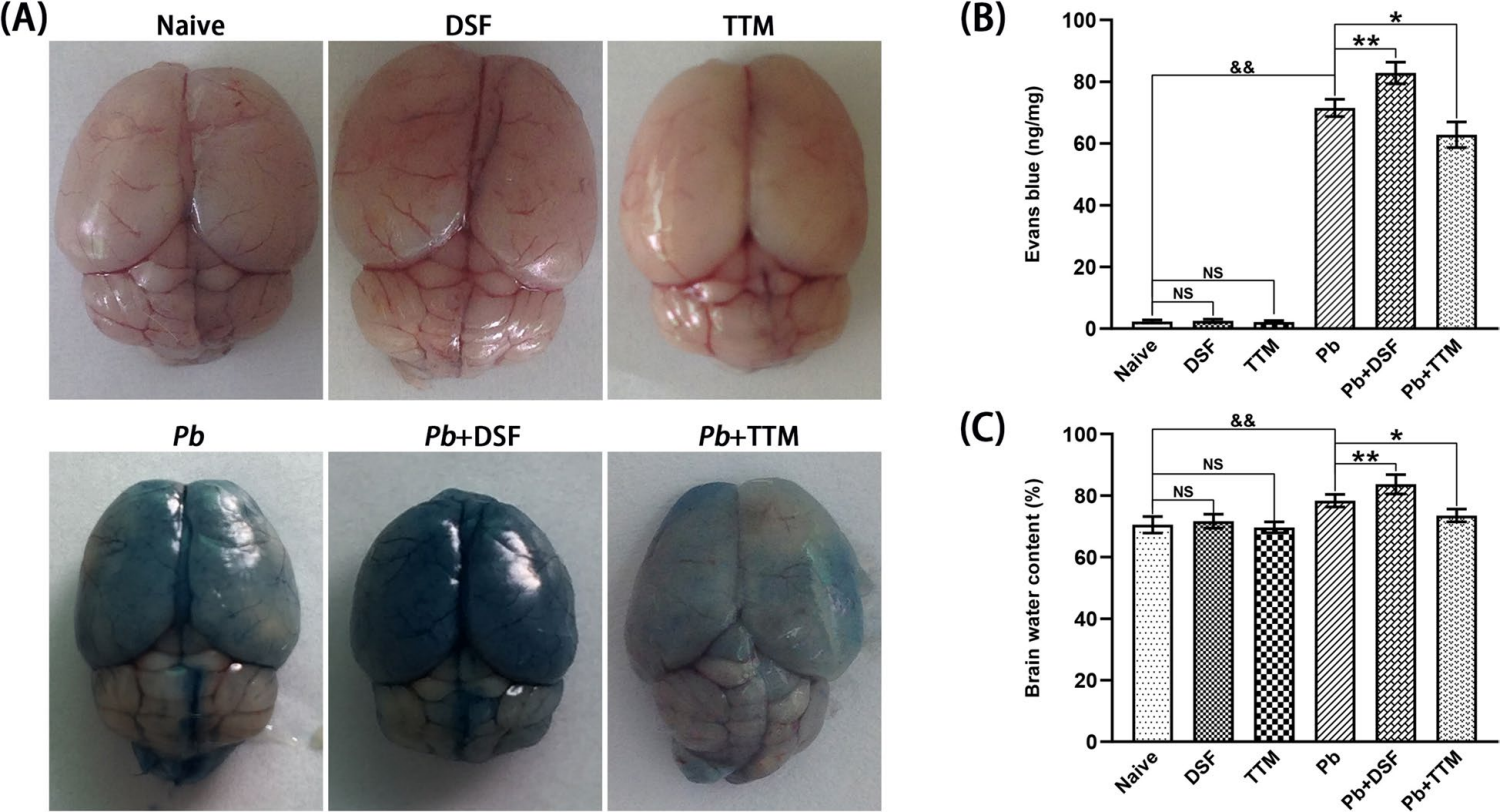
Effects of DSF or TTM treatment on BBB integrity and cerebral edema in ECM mice. **A** Representative images of Evans Blue-stained brain demonstrating BBB permeability across uninfected mice (naive, DSF, and TTM) or ECM mice (*Pb*, *Pb* + DSF, and *Pb* + TTM). **B** Quantification of Evans Blue extravasation expressed as nanogram dye per milligram tissue (ng/mg). **C** Cerebral edema assessment through brain water content measurement. Statistical analysis: Intergroup comparisons used independent *t* tests (two groups) or one-way ANOVA with Tukey’s post-hoc test (multi-group comparisons). Data represent mean ± SD (*n* = 6 mice/group; triplicate experiments). Significance thresholds: ^*^P < 0.05, ^**^P < 0.01 versus infected controls; ^&&^ *P* < 0.01 versus naive mice (group a); NS (not significant, *P* > 0.05) indicates no difference compared with naive mice

To evaluate the effects of DSF or TTM on BBB integrity in ECM mice, Evans Blue extravasation was quantified across experimental groups. *Pb*A infection induces inflammation, vascular damage, and toxin release, leading to BBB dysfunction and subsequent Evans Blue permeation into the brain parenchyma, visible as dark blue staining. As illustrated in Fig. 3A, no Evans Blue leakage was detected in uninfected control mice (naive, DSF-only, or TTM-only group). In contrast, ECM mice (*Pb*, *Pb* + DSF, and *Pb* + TTM groups) exhibited pronounced Evans Blue infiltration, consistent with BBB disruption. Notably, DSF treatment exacerbated Evans Blue accumulation in *Pb*A-infected mice compared with untreated ECM controls (82.8 ng/mg versus 71.5 ng/mg, *P* < 0.01), while TTM administration significantly attenuated dye penetration (62.8 ng/mg versus 71.5 ng/mg, *P* < 0.05). These data suggest divergent roles of DSF and TTM in modulating BBB permeability during ECM progression.

To assess the role of DSF or TTM in cerebral edema progression, brain tissue water content was measured across experimental groups. As shown in Fig. 3C, *Pb*A infection-induced ECM mice (*Pb*, *Pb* + DSF, and *Pb* + TTM groups) displayed significantly elevated cerebral water content compared with uninfected controls (*P* < 0.01). Notably, DSF-treated ECM mice exhibited a marked increase in brain water content relative to untreated ECM controls (83.7% versus 78.3%; *P* < 0.01), whereas TTM administration reduced cerebral edema in *Pb*A-infected mice (73.5% versus 78.3%; *P* < 0.05). These findings further corroborate that DSF exacerbates BBB dysfunction and potentiates brain edema, while TTM mitigates neuropathological damage in ECM.

### Effects of DSF or TTM treatment on neuroinflammatory responses in ECM mice

**Fig. 4:**
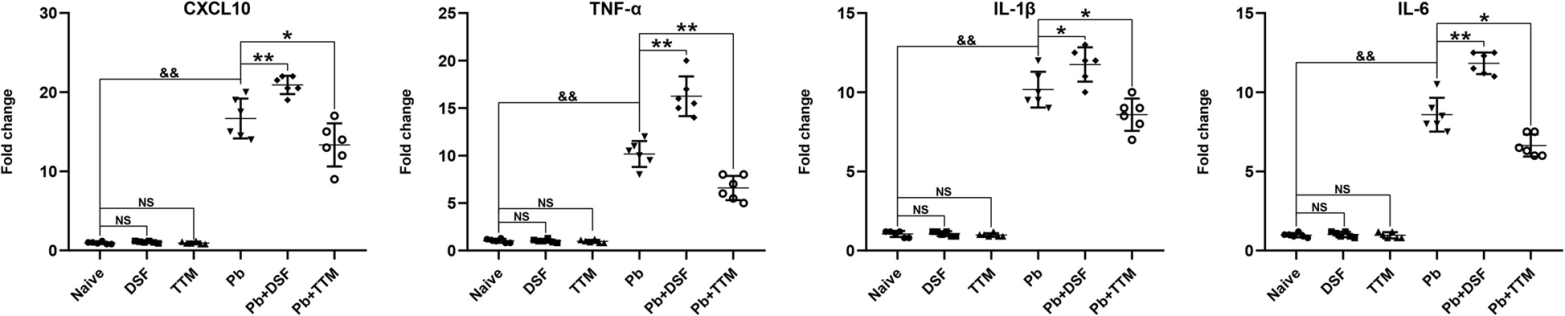
Effects of DSF or TTM treatment on neuroinflammatory responses in ECM mice. Total RNA was extracted from cerebral tissues of randomly selected uninfected mice (naive, DSF, and TTM; *n* = 6/group) or ECM mice (*Pb*, *Pb* + DSF, and *Pb* + TTM; *n* = 6/group), followed by reverse transcription. mRNA expression levels of CXCL10, tumor necrosis factor (TNF)-ɑ, interleukin (IL)-1β, and IL-6 were quantified via qPCR using the 2^−ΔΔCT^ method. Statistical analysis was performed using one-way ANOVA with post-hoc Tukey’s test for multiple comparisons. Data represent three independent experimental replicates (six mice per group per experiment) and are expressed as mean ± SD. ^*^P < 0.05 and ^**^P < 0.01 indicate significant differences compared with *Pb*-infected controls; ^&&^ *P* < 0.01 versus naive mice (group a); NS (not significant, *P* > 0.05) indicates no difference compared with naive mice

To investigate the modulatory effects of DSF or TTM on neuroinflammation in ECM, we quantified the mRNA levels of proinflammatory mediators (CXCL10, tumor necrosis factor (TNF)-α, interleukin (IL)-1β, and IL-6) in brain tissues via qPCR. As shown in Fig. 4, uninfected controls (naive, DSF-only, and TTM-only groups) exhibited basal expression levels of all cytokines, with no intergroup differences (*P* > 0.05). In contrast, the untreated ECM control mice (*Pb* group) displayed marked upregulation of CXCL10, TNF-α, IL-1β, and IL-6 compared with uninfected controls (*P* < 0.01). Strikingly, DSF treatment further amplified mRNA expression of CXCL10 (*P* < 0.01), TNF-α (*P* < 0.01), IL-1β (*P* < 0.05), and IL-6 (*P* < 0.01) relative to untreated ECM controls (*Pb* group). Conversely, TTM administration significantly suppressed these proinflammatory markers (CXCL10: *P* < 0.05; TNF-α: *P* < 0.01; IL-1β and IL-6: *P* < 0.05). Collectively, these data demonstrate that DSF exacerbates neuroinflammation in ECM, whereas TTM exerts potent antiinflammatory effects, aligning with their respective impacts on BBB integrity and cerebral edema.

### Effects of DSF or TTM treatment on astrocyte reactivity in ECM mice

**Fig. 5:**
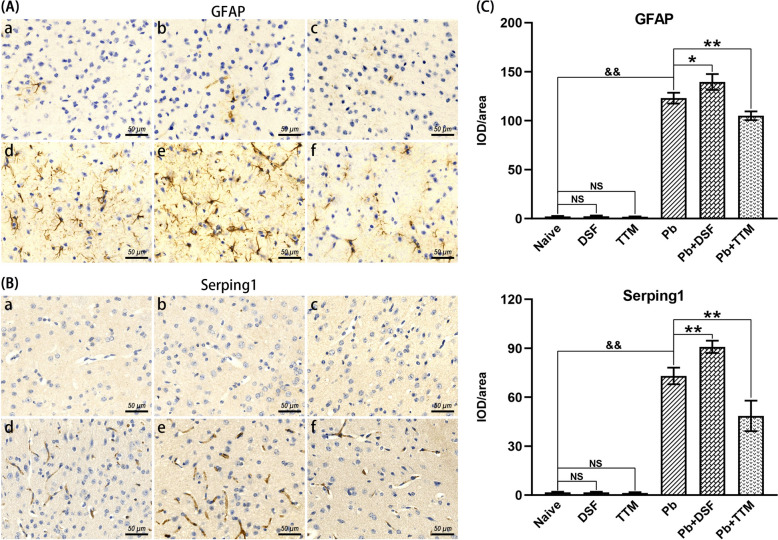
Effects of DSF or TTM treatment on astrocyte reactivity in ECM mice. **A, B** Representative immunohistochemical staining images of GFAP (astrocyte reactivity marker) and Serping1 (astrocyte reactivity marker) in the cerebral cortex captured under light microscopy (400 × magnification). Experimental groups: **a** naive mice; **b** DSF-treated uninfected controls; **c** TTM-treated uninfected controls; **d** untreated *Pb*A-infected mice; **e** DSF-treated infected mice; **f** TTM-treated infected mice. **C** Quantification of GFAP^+^ and Serping1^+^ astrocytes expressed as integrated optical density (IOD)/area. Data were obtained from > 20 fields per tissue slice, with six mice per group and three independent experimental replicates. Values represent mean ± standard deviation. Statistical significance: ^**^ *P* < 0.01 and ^*^ *P* < 0.05 versus infected control (group d); ^&&^* P* < 0.01 versus naive mice (group a); NS (not significant, *P* > 0.05) indicates no difference compared with naive mice

To evaluate the modulation of astrocyte reactivity by DSF or TTM during ECM progression, GFAP^+^ and Serping1^+^ (markers of astrocyte reactivity) astrocytes were quantified in cortical tissues via immunohistochemistry. As shown in Fig. 5, uninfected control groups (naive, DSF-only, and TTM-only) exhibited minimal GFAP^+^ and Serping1^+^ immunoreactivity (IOD/area), with no intergroup differences. In contrast, *Pb*A infection-induced ECM controls (*Pb* group) demonstrated a significant increase in GFAP^+^ (*P* < 0.01) and Serping1^+^ (*P* < 0.01) astrocytes compared with uninfected mice. Strikingly, DSF treatment further elevated astrocyte reactivity in ECM mice, with GFAP^+^ levels rising from 123.0 to 139.5 IOD/area (*P* < 0.05) and Serping1^+^ levels increasing from 73.0 to 90.8 IOD/area (*P* < 0.01). Conversely, TTM administration suppressed both GFAP^+^ (105.0 versus 123.0 IOD/area; *P* < 0.01) and Serping1^+^ (48.5 versus 73.0 IOD/area; *P* < 0.01) immunoreactivity. These results indicate that *Pb*A infection drives cortical astrocyte reactivity. Critically, DSF exacerbates this pathological activation, while TTM attenuates astrocyte reactivity, aligning with its neuroprotective effects observed in prior assays.

### Effects of DSF or TTM treatment on cerebral copper accumulation and cuproptosis in ECM mice

**Fig. 6:**
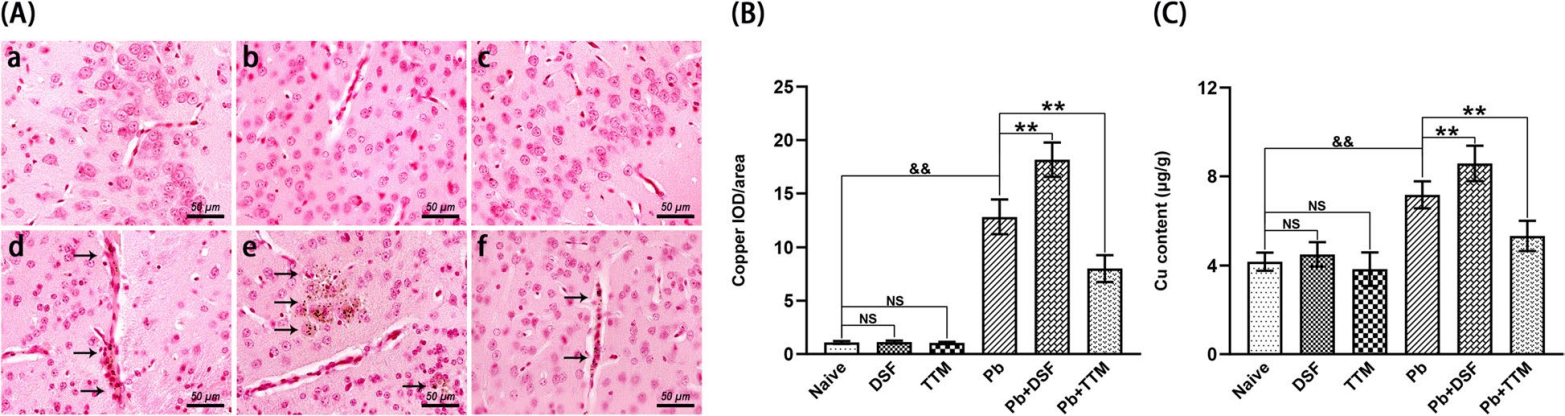
Effects of DSF or TTM treatment on cerebral copper accumulation in ECM mice. **A** Representative rubeanic acid copper (RAC) staining images of copper salt deposits in cortical brain tissue captured under light microscopy (400 × magnification). Experimental groups: **a** naive mice; **b** DSF-treated uninfected controls; **c** TTM-treated uninfected controls; **d** untreated *Pb*A-infected mice; **e** DSF-treated infected mice; **f** TTM-treated infected mice. **B** Quantitative analysis of copper granules expressed as integrated optical density (IOD)/area. **C** Total cerebral copper content quantified by inductively coupled plasma mass spectrometry (ICP-MS). Data were derived from six mice per group, with > 20 fields analyzed per tissue slice and three independent experimental replicates. Values represent mean ± standard deviation. Statistical significance: ^**^ *P* < 0.01 and ^*^ *P* < 0.05 versus infected control (group d); ^&&^ *P* < 0.01 versus naive mice (group a); NS (not significant, *P* > 0.05) indicates no difference compared with naive mice

To assess copper accumulation and cuproptosis in ECM pathogenesis, cortical brain tissues were analyzed via RAC staining and ICP-MS analysis. RAC staining (Fig. 6A, B) revealed negligible copper deposits in uninfected controls (naive, DSF-only, and TTM-only groups). In contrast, *Pb*A infecion-induced ECM controls (*Pb* group) exhibited pronounced copper accumulation compared with controls (*P* < 0.01). DSF treatment exacerbated this phenotype, with *Pb* + DSF ECM mice showing a significant increase in cortical copper deposits relative to the *Pb* group (*P* < 0.01), whereas TTM administration markedly reduced copper accumulation in *Pb* + TTM ECM mice (*P* < 0.01). Consistent with RAC findings, ICP-MS quantification of total cerebral copper (Fig. 6C) confirmed identical trends: DSF amplified (*P* < 0.01), and TTM suppressed (*P* < 0.01), copper overload in ECM mice. These data collectively demonstrate that DSF potentiates cuproptosis-associated copper accumulation, while TTM alleviates cerebral copper dyshomeostasis, suggesting a mechanistic link between copper regulation and ECM pathology.

**Fig. 7:**
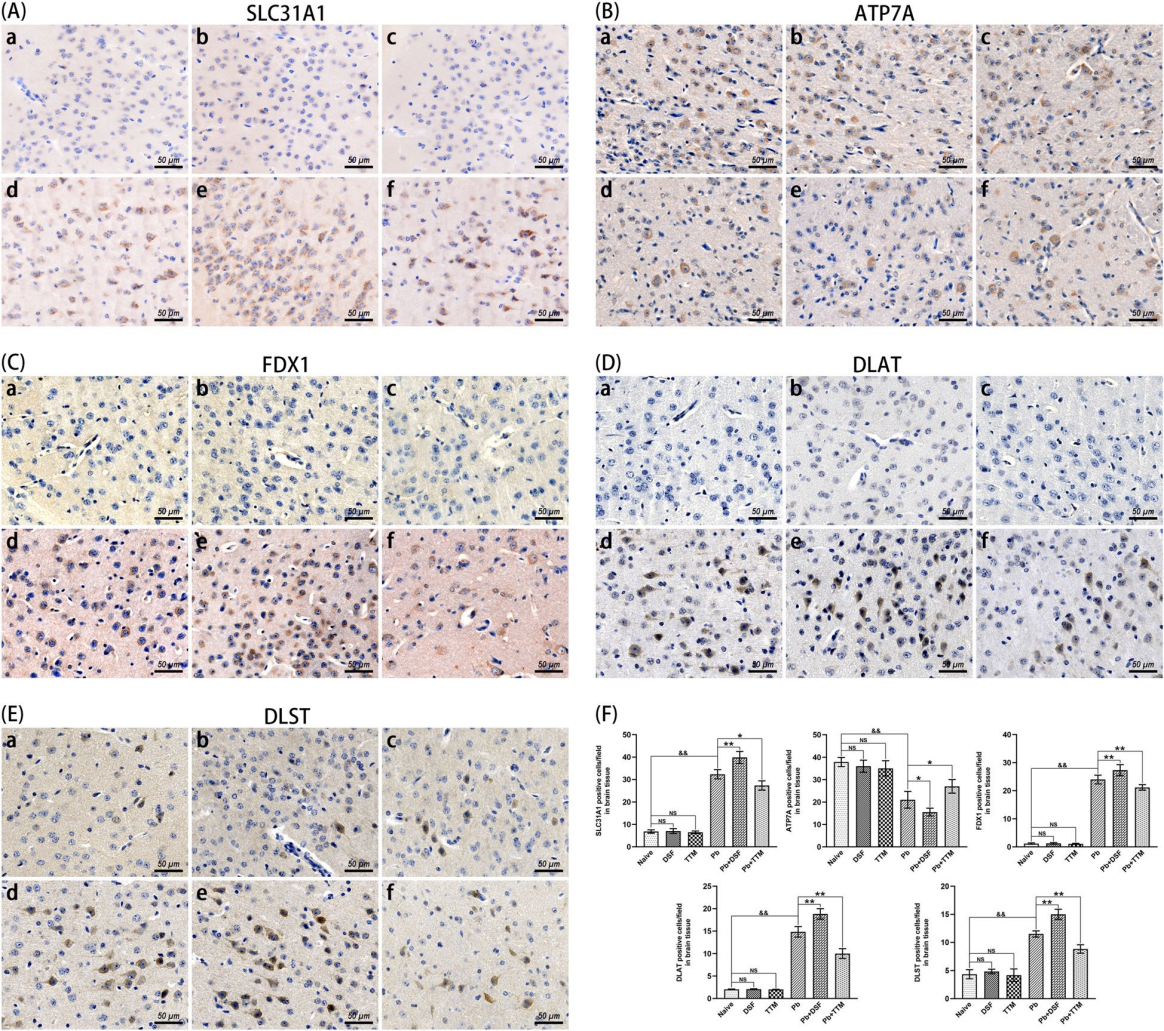
Effects of DSF or TTM treatment on astrocytic cuproptosis markers in ECM mice. **A**-**E** Representative immunohistochemical staining images of SLC31A1 (copper influx transporter), ATP7A (copper efflux transporter), FDX1 (key cuproptosis regulator), DLAT, and DLST (lipoylated precursor proteins) in the cerebral cortex captured under light microscopy (400 × magnification). Experimental groups: **a** naive mice; **b** DSF-treated uninfected controls; **c** TTM-treated uninfected controls; **d** untreated *Pb*A-infected mice; **e** DSF-treated infected mice; **f** TTM-treated infected mice. **F** Quantification of SLC31A1^+^, ATP7A^+^, FDX1^+^, DLAT^+^, and DLST^+^ cells expressed as number per field. Data were obtained from > 20 fields per tissue slice, with six mice per group and three independent experimental replicates. Values represent mean ± standard deviation. Statistical significance: ^**^ *P* < 0.01 and ^*^ *P* < 0.05 versus infected control (group d); ^&&^ *P* < 0.01 versus naive mice (group a); NS (not significant, *P* > 0.05) indicates no difference compared with naive mice

To investigate molecular mechanisms underlying cerebral cuproptosis, cortical tissues from unifected mice and ECM mice were analyzed for key cuproptosis regulators (SLC31A1, ATP7A, FDX1, DLAT, and DLST) via immunohistochemistry (Fig. 7). compared with uninfected controls (naive, DSF-only, TTM-only), *Pb*A infection-induced ECM controls (*Pb* group) exhibited upregulated expression of SLC31A1 (*P* < 0.01), FDX1 (*P* < 0.01), DLAT (*P* < 0.01), and DLST (*P* < 0.01), alongside downregulated ATP7A (*P* < 0.01). DSF treatment (*Pb* + DSF) further amplified these trends, elevating SLC31A1 (*P* < 0.01), FDX1 (*P* < 0.01), DLAT (*P* < 0.01), and DLST (*P* < 0.01), while suppressing ATP7A (*P* < 0.05) compared with the *Pb* group. Conversely, TTM administration (*Pb* + TTM) reversed this profile, reducing SLC31A1 (*P* < 0.05), FDX1 (*P* < 0.01), DLAT (*P* < 0.01), and DLST (*P* < 0.01), but enhancing ATP7A (*P* < 0.05). These findings demonstrate that ECM pathogenesis involves dysregulated cuproptosis signaling (SLC31A1↑/ATP7A↓-mediated copper overload and FDX1/DLAT/DLST↑-driven mitochondrial toxicity). Critically, DSF exacerbates this pathway, whereas TTM restores copper homeostasis and mitigates cuproptosis, aligning with its neuroprotective efficacy observed in prior analyses.

### Effects of DSF or TTM treatment on astrocytic cuproptosis markers in ECM mice

**Fig. 8:**
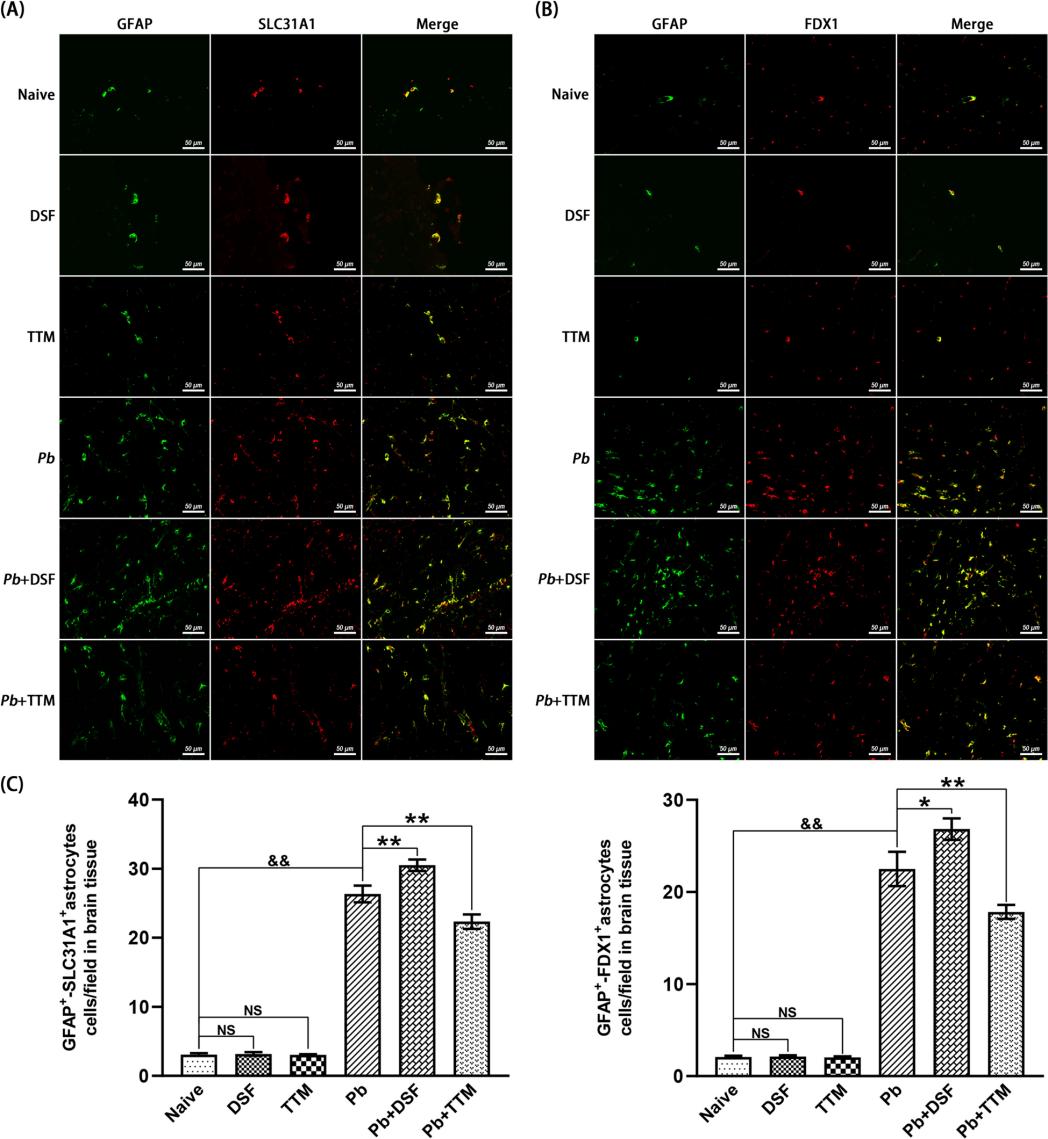
Effects of DSF or TTM treatment on GFAP^+^-SLC31A1^+^ and GFAP^+^-FDX1^+^ astrocytes in the cerebral cortex of ECM mice. **A**, **B** Representative double immunofluorescence staining images of GFAP^+^-SLC31A1^+^ and GFAP^+^-FDX1^+^ astrocytes in the cerebral cortex captured under fluorescence microscopy (400 × magnification). Experimental groups: **a** naive mice; **b** DSF-treated uninfected controls; **c** TTM-treated uninfected controls; **d** untreated *Pb*A-infected mice; **e** DSF-treated infected mice; **f** TTM-treated infected mice. GFAP^+^ astrocytes (green fluorescence), SLC31A1^+^ or FDX1^+^ cells (red fluorescence), and co-localized GFAP^+^-SLC31A1^+^ or GFAP^+^-FDX1^+^ astrocytes (merged signal, yellow) are shown. **C** Quantification of GFAP^+^-SLC31A1^+^ and GFAP^+^-FDX1^+^ astrocytes expressed as number per field. Data were derived from > 20 fields per tissue slice, with six mice per group and three independent experimental replicates. Values represent mean ± standard deviation. Statistical significance: ^**^ *P* < 0.01 and ^*^ *P* < 0.05 versus *Pb*A-infected control mice (group d); ^&&^ *P* < 0.01 versus naive mice (group a); NS (not significant,* P* > 0.05) indicates no difference compared with naive mice

**Fig. 9:**
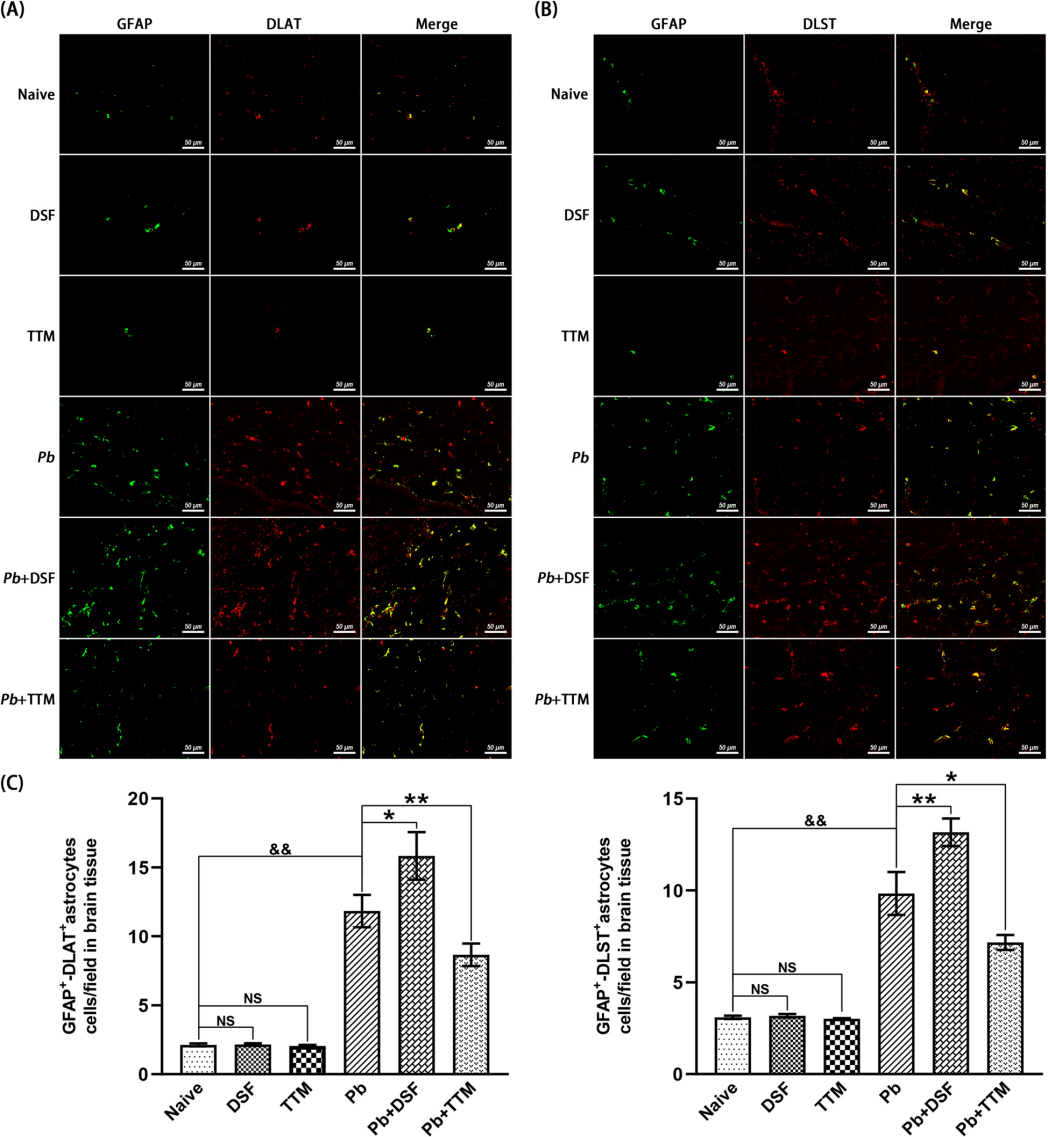
Effects of DSF or TTM treatment on GFAP^+^-DLAT^+^ and GFAP^+^-DLST^+^ astrocytes in the cerebral cortex of ECM mice. **A**, **B** Representative double immunofluorescence staining images of GFAP^+^-DLAT^+^ and GFAP^+^-DLST^+^ astrocytes in the cerebral cortex captured under fluorescence microscopy (400 × magnification). Experimental groups: **a** naive mice; **b** DSF-treated uninfected controls; **c** TTM-treated uninfected controls; **d** untreated *Pb*A-infected mice; **e** DSF-treated infected mice; **f** TTM-treated infected mice. GFAP^+^ astrocytes (green fluorescence), DLAT^+^ or DLST^+^ cells (red fluorescence), and colocalized GFAP^+^-DLAT^+^ or GFAP^+^-DLST^+^ astrocytes (merged signal, yellow) are shown. **C** Quantification of GFAP^+^-DLAT^+^ and GFAP^+^-DLST^+^ astrocytes expressed as number per field. Data were derived from > 20 fields per tissue slice, with six mice per group and three independent experimental replicates. Values represent mean ± standard deviation. Statistical significance: ^**^ *P* < 0.01 and ^*^ *P* < 0.05 versus *Pb*A-infected control mice (group d); ^&&^ *P* < 0.01 versus naive mice (group a); NS (not significant,* P* > 0.05) indicates no difference compared with naive mice

To determine the astrocyte-specific regulation of cuproptosis, colocalization of GFAP (pan-astrocyte marker) with SLC31A1, FDX1, DLAT, or DLST was quantified in cortical tissues (Figs. 8, 9). Uninfected controls (naive, DSF-only, TTM-only) exhibited minimal GFAP^+^/SLC31A1^+^, GFAP^+^/FDX1^+^, GFAP^+^/DLAT^+^, and GFAP^+^/DLST^+^ colabeled astrocytes. *Pb*A infection-induced ECM controls (*Pb* group) showed marked increases in all colabeled populations compared with controls (*P* < 0.01 for each). DSF treatment (*Pb* + DSF) amplified this response, elevating GFAP^+^/SLC31A1^+^ (30.5 versus 26.3 cells/field; *P* < 0.01), GFAP^+^/FDX1^+^ (26.8 versus 22.5 cells/field; *P* < 0.05), GFAP^+^/DLAT^+^ (15.8 versus 11.8 cells/field;* P* < 0.05), and GFAP^+^/DLST^+^ (13.2 versus 9.8 cells/field; *P* < 0.01) counts relative to the *Pb* group. Conversely, TTM administration (*Pb* + TTM) suppressed these populations: GFAP^+^/SLC31A1^+^ (22.3 versus 26.3 cells/field; *P* < 0.01), GFAP^+^/FDX1^+^ (17.8 versus 22.5 cells/field; *P* < 0.01), GFAP^+^/DLAT^+^ (8.7 versus 11.8 cells/field; *P* < 0.01), and GFAP^+^/DLST^+^ (7.2 versus 9.8 cells/field; *P* < 0.05). These results confirm that ECM induces astrocytic upregulation of copper uptake (SLC31A1^+^) and mitochondrial cuproptosis effectors (FDX1^+^/DLAT^+^/DLST^+^), with DSF exacerbating and TTM mitigating this astrocyte-specific cuproptotic cascade.

### Effects of DSF-CuCl_2_ or TTM-CuCl_2_ on viability of *Pb*Ag-stimulated astrocyte in vitro

**Fig. 10:**
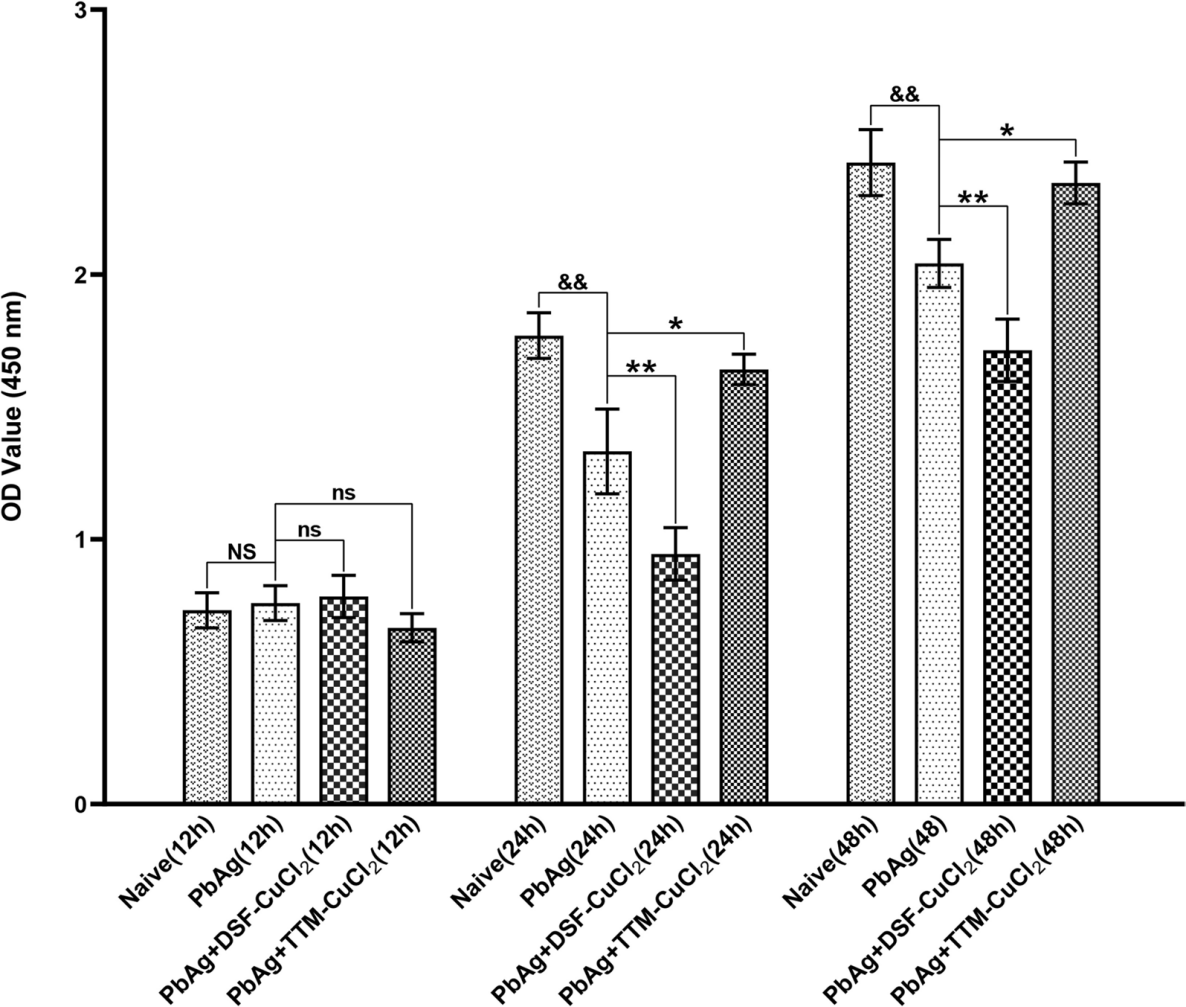
Effects of DSF-CuCl_2_ or TTM-CuCl_2_ on astrocyte vialibity upon *Pb*Ag-stimulation in vitro using CCK-8 assay. The astrocytes were incubated with equal volume PBS, *Pb*Ag (20 μg/mL), *Pb*Ag (20 μg/mL) + DSF (20 nM), or *Pb*Ag (20 μg/mL) + TTM (20 nM) for 12 h, 24 h, or 48 h, respectively. Following treatment, the cells were incubated with CCK-8, and the absorbance of each well was measured at 450 nm to assess cell viability. Data were derived from three independent experiments and expressed as mean ± standard deviation. Statistical significance: ^&^ *P* < 0.05 and ^&&^ *P* < 0.01 versus only PBS-treated astrocytes (naive group) at 24 or 48 h, respectively; ^*^ *P* < 0.05 and ^**^ *P* < 0.01 versus only *Pb*Ag-stimulated astrocytes (*Pb*Ag group) at 24 or 48 h, respectively; NS (not significant,* P* > 0.05) indicates no difference compared with only PBS-treated astrocytes; ns (not significant,* P* > 0.05) indicates no difference compared with only *Pb*Ag-stimulated astrocytes

To investigate the impact of copper-modulating agents on the viability of astrocytes stimulated with *Pb*Ag in vitro, astrocytes were exposed to *Pb*Ag and subsequently treated with DSF-CuCl_2_ or TTM-CuCl_2_; viability was assessed using the CCK-8 assay at 24 or 48 h (Fig. 10). Astrocytes viability among the unstimulated control (naive), *Pb*Ag-stimulated *(Pb*Ag), *Pb*Ag-stimulated plus DSF-CuCl_2_ (*Pb*Ag + DSF-CuCl_2_), and *Pb*Ag-stimulated plus TTM-CuCl_2_ (*Pb*Ag + TTM-CuCl_2_) groups showed no significant differences at 24 h. Compared with the naive group, *Pb*Ag stimulation significantly reduced cell viability, as indicated by OD values decreased by ~24.7% (*P* < 0.01) at 24 h and ~15.7% (*P* < 0.01) at 48 h. Treatment with DSF-CuCl_2_ further potentiated this *Pb*Ag-induced reduction in viability, decreasing OD values by an additional ~29.1% (*P* < 0.01) at 24 h and ~16.1% (*P* < 0.01) at 48 h relative to the *Pb*Ag group alone. Conversely, treatment with TTM-CuCl_2_ significantly attenuated the *Pb*Ag-induced viability reduction, increasing OD values by ~23.2% (*P* < 0.05) at 24 h and ~14.8% (*P* < 0.05) at 48 h compared with the *Pb*Ag group. These results indicate that DSF-CuCl_2_ treatment exacerbates the *Pb*Ag-induced decrease in astrocyte viability, whereas TTM-CuCl_2_ treatment mitigates this reduction.

### Effects of DSF-CuCl_2_ or TTM-CuCl_2_ on cuproptosis and astrocyte reactivity in iRBCs-stimulated astrocytes

**Fig. 11:**
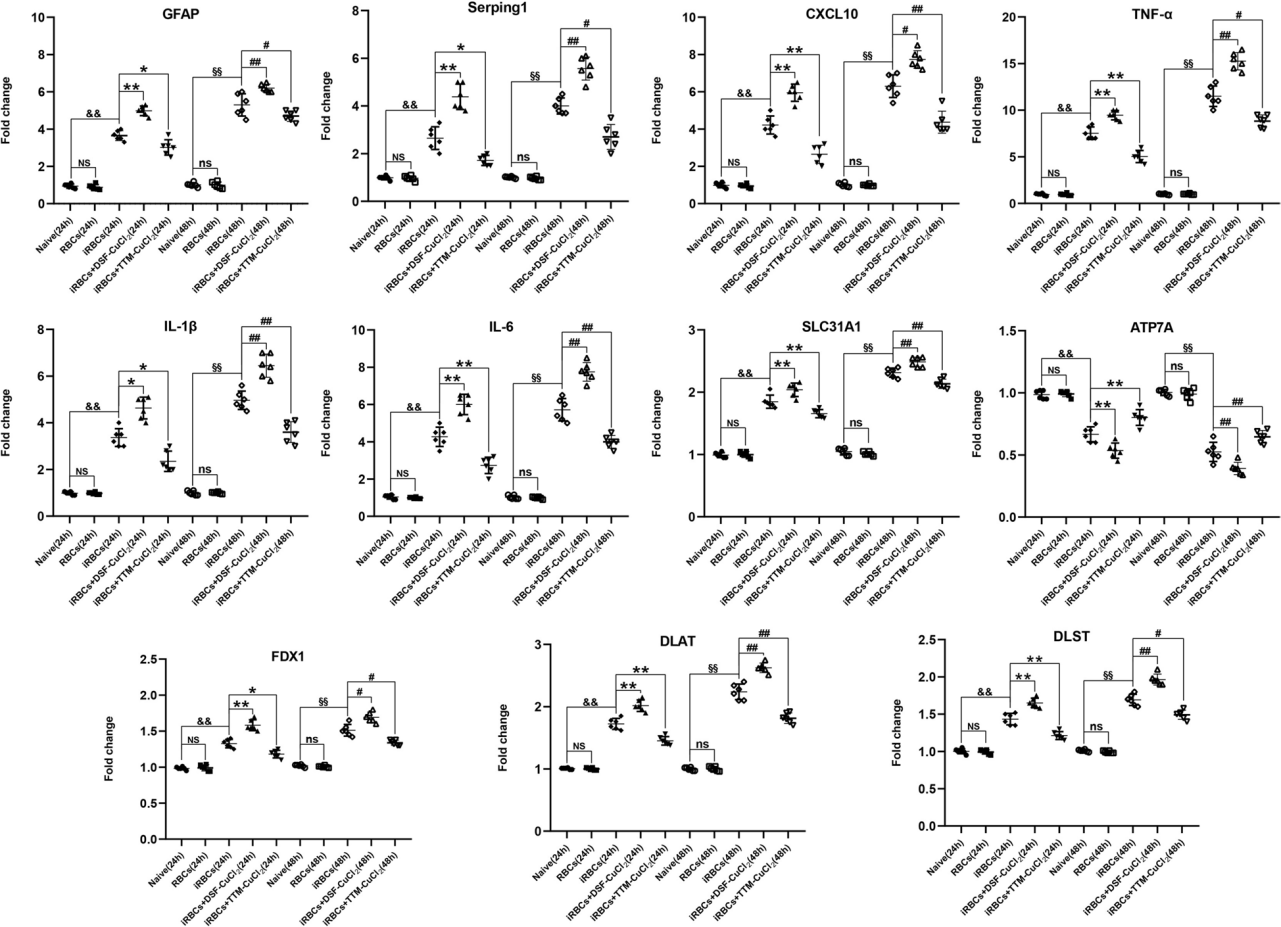
Effects of DSF-CuCl_2_ or TTM-CuCl_2_ coculture on mRNA expression in iRBCs-stimulated astrocytes in vitro. Total RNA was isolated from astrocytes after coculture under the following conditions: **a** PBS-treated control (24 h or 48 h); **b** nonparasitized red blood cells (RBCs)-treated astrocytes (24 h or 48 h); **c** iRBCs-stimulated astrocytes (24 h or 48 h); **d** iRBCs-stimulated astrocytes cocultured with DSF-CuCl_2_ (24 h or 48 h); **e** iRBCs-stimulated astrocytes cocultured with TTM-CuCl_2_ (24 h or 48 h). mRNA levels of GFAP, Serping1, CXCL10, TNF-α, IL-1β, IL-6, SLC31A1, ATP7A, FDX1, DLAT, and DLST were quantified by qPCR using the 2^−ΔΔCT^ method. Data were derived from three independent experiments and expressed as mean ± standard deviation. Statistical significance: ^&^ *P* < 0.05 and ^&&^ *P* < 0.01, or ^§^ *P* < 0.05 and ^§§^ *P* < 0.01 versus PBS-treated astrocytes at 24 or 48 h, respectively; ^*^ *P* < 0.05 and ^**^ *P* < 0.01, or ^#^ *P* < 0.05 and ^##^ *P* < 0.01 versus iRBCs-stimulated astrocytes at 24 or 48 h, respectively; NS (not significant,* P* > 0.05) or ns (not significant,* P* > 0.05) indicates no difference compared with PBS-stimulated astrocytes at 24 or 48 h, respectively

To validate the role of copper homeostasis in astrocytic responses to *Pb*A-iRBCs, astrocytes in cocultured with *Pb*A-iRBCs were treated with DSF-CuCl_2_ or TTM-CuCl_2_ for 24 or 48 h (Fig. 11). qPCR analysis demonstrated that stimulation with non-parasitized RBCs did not significantly alter the expression levels of astrocyte reactivity markers (GFAP and Serping1), proinflammatory cytokines (CXCL10, TNF-α, IL-1β, and IL-6), or cuproptosis regulators (SLC31A1, ATP7A, FDX1, DLAT, DLST) compared with unstimulated controls (*P* > 0.05 for all genes). In contrast, the *Pb*A-iRBCs stimulation significantly upregulated mRNA levels of astrocyte reactivity markers (GFAP and Serping1), proinflammatory cytokines (CXCL10, TNF-α, IL-1β, and IL-6), and cuproptosis regulators (SLC31A1, FDX1, DLAT, and DLST), while suppressing ATP7A expression (*P* < 0.01 versus naive at both time points). Treatment with DSF-CuCl_2_ amplified these *Pb*A-iRBCs-induced effects, further elevating GFAP (*P* < 0.01), Serping1 (*P* < 0.01), CXCL10 (*P* < 0.05/0.01), TNF-α (*P* < 0.01), IL-1β (*P* < 0.05/0.01), IL-6 (*P* < 0.01), SLC31A1 (*P* < 0.01), FDX1 (*P* < 0.05/0.01), DLAT (*P* < 0.01), and DLST (*P* < 0.01), while reducing ATP7A (*P* < 0.01). Conversely, TTM-CuCl_2_ treatment counteracted the changes induced by *Pb*A-iRBCs, suppressing GFAP (*P* < 0.05), Serping1 (*P* < 0.05), CXCL10 (*P* < 0.01), TNF-α (*P* < 0.05/0.01), IL-1β (*P* < 0.05/0.01), IL-6 (*P* < 0.01), SLC31A1 (*P* < 0.01), FDX1 (*P* < 0.05), DLAT (P < 0.01), and DLST (*P* < 0.05/0.01), while restoring ATP7A (*P* < 0.01) expression. Collectively, these data demonstrate that DSF-CuCl_2_ synergizes with *Pb*A-iRBCs to drive a pathogenic cascade characterized by copper overload (SLC31A1↑/ATP7A↓), mitochondrial cuproptosis (FDX1/DLAT/DLST↑), and inflammatory astrocyte reactivity, whereas TTM-CuCl_2_ reverses this pathogenic cascade by restoring copper homeostasis.

## Discussions

Cuproptosis, a recently defined programmed cell death pathway distinct from canonical apoptosis or necrosis, is driven by copper overload-induced proteotoxic stress through lipoylated protein aggregation and iron-sulfur cluster depletion [25]. Clinical studies have consistently reported elevated serum copper levels in patients with malaria [27, 28, 37], with emerging evidence linking copper dyshomeostasis to astrocyte reactivity-induced neuroinflammatory [38, 39]. Cerebral malaria (CM), a lethal complication of *P. falciparum* infection, is characterized by BBB breakdown, neuroinflammation, and microhemorrhages [3, 6]. Reactive astrocytes are recognized contributors to BBB disruption in CM pathogenesis [19], yet the upstream triggers of this reactivity remain poorly defined. Our study provides the novel evidence that cuproptosis mediates astrocyte-driven neuropathology in ECM. Using a *Pb*A-infected C57BL/6 mouse model, we demonstrated significant cerebral copper accumulation and upregulated cuproptosis markers in ECM progression. Pharmacological modulation revealed a causal relationship: DSF exacerbated copper overload (SLC31A1↑/ATP7A↓), amplified mitochondrial cuproptosis (FDX1/DLAT/DLST↑), and intensified astrocyte reactivity, thereby worsening ECM severity. Conversely, TTM restored copper homeostasis, suppressed cuproptotic signaling, and attenuated neuroinflammation. These findings identify astrocytic cuproptosis as a critical mechanism in CM pathogenesis and propose copper chelation as a novel therapeutic strategy to mitigate BBB disruption and neurological sequelae.

Accumulating evidence indicates that cuproptosis contributes to the pathogenesis of various brain diseases [39–42, 43]. Notably, serum copper levels are reduced in patients with uncomplicated malaria [33, 44] but elevated in cerebral malaria (CM) cases [45, 46], with further increases observed during the acute phase of malaria infection [47]. However, the mechanistic role of cuproptosis in CM remained unexplored. In this study, we demonstrated significant copper accumulation in the brain tissues of ECM mice compared with uninfected controls, accompanied by upregulated expression of cuproptosis-associated genes SLC31A1 (copper influx transporter), FDX1 (key regulatory factor), DLAT, and DLST (lipoylated proteins), alongside downregulated ATP7A (copper efflux transporter). These findings were corroborated in vitro, where iRBCs-stimulated astrocytes exhibited similar transcriptional changes. Our data align with prior reports showing that disulfiram (DSF), a copper ionophore, enhances cuproptosis, whereas the chelator tetrathiomolybdate (TTM) suppresses it [25, 48–50]. To validate this mechanism in ECM, DSF-treated mice exhibited aggravated cerebral pathology, elevated parasite burden, increased copper deposition, and dysregulated cuproptosis-related gene expression, whereas TTM ameliorated these effects. Parallel in vitro experiments revealed that DSF-CuCl_2_ amplified cuproptosis-related gene dysregulation in astrocytes, while TTM-CuCl_2_ reversed it. These results are consistent with studies linking cuproptosis to neurological disorders via pathways such as HSP70–TLR4–NLRP3-mediated inflammation [51], CREB–BDNF-dependent synaptic dysfunction [31], and glioma progression [52]. Furthermore, cuproptosis-related genes (LIPT1, FDX1, and DLAT) have emerged as therapeutic targets in epilepsy and gliomas [53, 54], underscoring its broad relevance. Collectively, our findings establish cuproptosis as a critical contributor to ECM pathogenesis and highlight its potential as a therapeutic target.

Astrocytic copper homeostasis imbalance plays a critical role in disease pathogenesis. Dysregulated copper transport may drive astrocyte demyelination in multiple sclerosis [55], while excess copper induces cytotoxicity by impairing mitochondrial function, reducing membrane potential, elevating reactive oxygen species (ROS), and disrupting glutathione (GSH) metabolism [56]. Copper chelation in Zika virus-infected astrocytes mitigates reactive oxygen species (ROS) and enhances viability [57], though paradoxically, copper may also induce oxidative stress-mediated neuroprotection alongside FDX1-independent astrocyte toxicity [34]. During CNS injury, astrocytes transition to a reactive state, with inflammatory astrocytes promoting neuroinflammation via proinflammatory cytokines, a process driven by microglial cytokines (e.g., IL-1α, TNF, and C1q) [13, 14]. Inflammatory astrocytes are implicated in neurodegenerative diseases, including multiple sclerosis, Alzheimer’s disease, and Parkinson’s disease [58], though transforming growth factor (TGF)β3 and N-acetylcysteine-conjugated dendrimers (D-NAC) can suppress proinflammatory response and improve neuronal survival [59, 60]. While reactive astrocytes are key contributors to CM pathogenesis [19, 61], the role of copper homeostasis in their reactivity during ECM remained unclear. In this study, DSF treatment exacerbated ECM severity in mice, increasing GFAP^+^ and Serping1^+^ astrocytes and upregulating proinflammatory cytokines (CXCL10, TNF-α, IL-1β, and IL-6) in both brain tissues and iRBCs-stimulated astrocytes. Conversely, TTM reversed these effects. Costaining revealed elevated GFAP^+^-SLC31A1^+^, GFAP^+^-FDX1^+^, GFAP^+^-DLAT^+^, and GFAP^+^-DLST^+^ astrocytes in DSF-treated ECM mice, consistent with reports linking copper overload (e.g., via FDX1/SLC31A1 upregulation) to glioma progression [62, 63] and copper/zinc ionophores (e.g., CPT/ZPT) to astrocyte toxicity [64]. Low-dose copper exposure similarly amplifies astrocyte reactivity and neurobehavioral deficits in ApoE4 mice [65], while antioxidants and chelators counteract copper-induced cytotoxicity [66]. Critically, our CCK-8 data also demonstrated that copper overload (DSF-CuCl_2_) potentiated PbAg-induced astrocyte death, while copper chelation (TTM-CuCl_2_) enhanced survival. These findings demonstrate that DSF promotes astrocyte reactivity via copper overload, aggravating ECM pathology, whereas TTM inhibits astrocyte reactivity and ameliorates disease severity.

While this study establishes the impact of DSF and TTM on brain tissue injury in ECM mice, several unresolved questions remain: (1) The mechanism underlying copper accumulation following *Pb*A infection remains uncharacterized; (2) The potential involvement of antiinflammatory astrocytes in cuproptosis during ECM pathogenesis was not investigated [67]; (3) The causal relationship between cuproptosis and astrocyte reactivity—specifically, how cuproptosis drives this reactivity—was not conclusively delineated by our data; (4) It is important to note that our in vivo analyses were conducted on brain tissues collected from ECM upon presentation of neurological symptoms, which occurred between days 6 and 9 postinfection. While this ensures we capture the acute pathological phase, it introduces a degree of temporal variability. Future studies employing fixed time-point analyses will be valuable to precisely delineate the kinetic profile of cuproptosis induction relative to other pathological events in ECM. Addressing these gaps will be critical to fully elucidate the role of cuproptosis in ECM progression and to inform therapeutic strategies targeting copper homeostasis.

## Conclusions

This study establishes cuproptosis as a contributor to ECM pathogenesis. We demonstrate significant copper accumulation, elevated cuproptosis-associated biomarkers, and increased proportions of GFAP^+^ astrocytes co-expressing SLC31A1, FDX1, DLAT, and DLST in ECM mouse brains. Functional studies revealed that DSF exacerbates neuropathology by promoting astrocyte reactivity and proinflammatory cytokine production, whereas TTM ameliorates these effects by suppressing reactivity and inflammation. While the precise mechanism linking cuproptosis to astrocyte reactivity in ECM progression requires further investigation, our findings identify astrocytic copper overload as a driver of neuroinflammation in ECM. Importantly, targeting cerebral cuproptosis—via inhibition of copper overload or modulation of its downstream effectors—may represent a novel therapeutic strategy to mitigate brain injury in CM.

## Supplementary Information

Supplementary Material 1. Primer sequences for qPCR analysis of target cytokines. *F* Forward primer, *R* Reverse primer. Primers were designed and synthesized by Sangon Biotech (Shanghai, China).

## Data Availability

Data supporting the main conclusions of this study are included in the manuscript.
