# Supplementary material for: Cuproptosis-driven astrocyte reactivity exacerbates experimental cerebral malaria pathogenesis
Source: Parasit Vectors. 2025 Nov 10;18:454. doi: 10.1186/s13071-025-07107-0 (PMC12604326; doi:10.1186/s13071-025-07107-0)
Supplement: Supplementary file 1 — Supplementary Material 1. Primer sequences for qPCR analysis of target cytokines. F Forward primer, R Reverse primer. Primers were designed and synthesized by Sangon Biotech (Shanghai, China). [file 13071_2025_7107_MOESM1_ESM.docx]

| Genes | Primers ^a^ |
| --- | --- |
| GFAP | F: 5’-TGCCACGCTTCTCCTTGTCTC-3’  R: 5’-CTCGCTCGCCCGTGTCTC-3’ |
| Serping1 | F: 5’-ACAGTGCTGCTAACTTAGAACTCATC-3’  R: 5’-ACGAGGCAGGTGTCAGAAGG-3’ |
| CXCL10 | F: 5’-GCCTCATCCTGCTGGGTCTG-3’  R: 5’-TTCCCTATGGCCCTCATTCTCAC-3’ |
| TNF-α | F: 5’-GCCTCTTCTCATTCCTGCTTGTGG-3’  R: 5’-GTGGTTTGTGAGTGTGAGGGTCTG-3’ |
| IL-1β | F: 5’-TCGCAGCAGCACATCAACAAG-3’  R: 5’-TCCACGGGAAAGACACAGGTAG-3’ |
| IL-6 | F: 5’-GAGAGGAGACTTCACAGAGGATACC-3’  R: 5’-TCATTTCCACGATTTCCCAGAGAAC-3’ |
| SLC31A1 | F: 5’-AACCACACGGACGACAACATTAC-3’  R: 5’-AAGTAGAAGGTCATAGGCATCATCATC-3’ |
| ATP7A  FDX1 | F: 5’-TGGCAAGGCAGAAGTAAGATATAACC-3’  R: 5’-TCATTCCTCTCACAACAAGTTCCAAG-3’  F: 5’-CGGAGCGGGAACTGCCATC-3’  R: 5’-TCTCGCCATCTCGGTTCTTGAAG-3’ |
| DLAT | F: 5’-CTCAGGCATGTATTCTGGCAATCG-3’  R: 5’-TCCAACTGCTCCATCCACAACTC-3’ |
| DLST  *Pb*A 18S rRNA | F: 5’-GGGTCTCGTGGTTCCTGTCATC-3’  R: 5’-TCCGTTGCTGATGGTGAAGGTAC-3’  F: 5’-ACGGGGAGCAAGAGCAGTATTTC-3’  R: 5’-CCCCACCAAAACCTGCCTTTATTG-3’ |
| GAPDH | F: 5’-GGCAAATTCAACGGCACAGTCAAG-3’  R: 5’-TCGCTCCTGGAAGATGGTGATGG-3’ |

**Supplementary Table 1. Primer sequences of target cytokines used for qPCR assay.**

Notes: ^a^ F, Forward primer; R, Reverse primer; Primers were designed and synthesized by Sangon Biotech (Shanghai, China).
